# Supplementary material for: Multiple factors affect discrimination learning performance, but not between-individual variation, in wild mixed-species flocks of birds
Source: R Soc Open Sci. 2020 Apr 29;7(4):192107. doi: 10.1098/rsos.192107 (PMC7211855; doi:10.1098/rsos.192107)
Supplement: Supplementary figures and tables [file rsos192107supp1.docx]

Supplementary Data for:

Multiple factors affect discrimination learning performance, but not between-individual variation, in wild mixed-species flocks of birds

Michael S. Reichert, Sam J. Crofts, Gabrielle L. Davidson, Josh A. Firth, Ipek G. Kulahci, John L. Quinn

Royal Society Open Science

Supplementary Table 1. Mean (SD) learning speed and number of visits for each experiment.

|  | Initial learning | | First reversal learning | | Second reversal learning | |
| --- | --- | --- | --- | --- | --- | --- |
|  | Avg (SD) learning speed | Avg (SD) N Visits | Avg (SD) learning speed | Avg (SD) N Visits | Avg (SD) learning speed | Avg (SD) N Visits |
| Blue tit female adult | 93.0 (147.6) | 423.5 (141.1) | 62.5 (83.2) | 452.1 (87.2) | 53.7 (60.2) | 438.5 (114.1) |
| Blue tit female juvenile | 58.9 (99.7) | 392.2 (162.5) | 51.1 (57.6) | 504.9 (138.2) | 60.5 (64.9) | 567.9 (91.2) |
| Blue tit male adult | 55.5 (93.8) | 390.6 (111.7) | 48.6 (46.4) | 448.3 (110.7) | 50.5 (56.7) | 482.5 (145.9) |
| Blue tit male juvenile | 75.8 (113.5) | 477.0 (154.5) | 64.1 (65.9) | 539.0 (127.7) | 64.4 (75.5) | 539.9 (132.1) |
| Great tit female adult | 19.5 (19.2) | 385.9 (149.9) | 87.0 (120.5) | 455.5 (113.7) | 109.8 (124.0) | 514.3 (121.5) |
| Great tit female juvenile | 30.7 (32.0) | 360.8 (193.1) | 73.2 (44.2) | 458.6 (147.9) | 118.0 (128.2) | 607.3 (207.3) |
| Great tit male adult | 39.6 (74.4) | 378.9 (127.5) | 36.9 (38.5) | 456.9 (95.6) | 60.6 (145.8) | 480.2 (158.0) |
| Great tit male juvenile | 21.6 (37.7) | 413.7 (124.9) | 44.7 (38.6) | 480.6 (95.4) | 104.0 (149.9) | 612.4 (217.4) |

Sample sizes are given in Table 1 in the main text. Average number of visits is calculated only from those birds that met the learning criterion.

Supplementary Table 2. Factors affecting participation in the initial learning experiment.

| fixed terms | estimate ± SE | *z* | *P* |
| --- | --- | --- | --- |
| Intercept | 0.66±0.25 |  |  |
| Age | **-0.73±0.27** | **-2.72** | **0.007** |
| Species | -0.16±0.35 | -0.44 | 0.66 |
| Species*Age | **0.91±0.45** | **2.03** | **0.043** |
| Feeder location | 0.21±0.21 | 1.00 | 0.32 |
| Sex | -0.16±0.21 | -0.75 | 0.45 |
| Age*Sex | 0.41±0.44 | 0.92 | 0.36 |
| Sex*Species | 0.34±0.44 | 0.78 | 0.44 |
| Age*Sex*Species | -0.61±0.91 | -0.66 | 0.51 |
|  |  |  |  |

Estimates, z values and P values are shown from a generalized linear mixed model (*N* = 409 individuals). Significant factors from the final model are shown in bold. The reference levels for categorical factors are as follows: age (juvenile), sex (female), species (blue tit), feeder location (centre).

Supplementary Table 3. Demographic effects of meeting participation and learning criterion in all experiments.

| Fixed terms | Estimate ± SE | *z* | *P* |
| --- | --- | --- | --- |
| Intercept | 0.14±0.35 |  |  |
| Age | -0.48±0.43 | -1.14 | 0.26 |
| Sex | -0.07±0.40 | -0.17 | 0.87 |
| Species | -0.78±0.53 | -1.47 | 0.14 |
| Age*Sex | -0.09±0.55 | -0.17 | 0.87 |
| Age*Species | 1.09±0.68 | 1.62 | 0.11 |
| Sex*Species | 0.31±0.71 | 0.43 | 0.67 |
| Age*Sex*Species | -0.51±0.90 | -0.57 | 0.57 |

Estimates, z values and P values are shown from a generalized linear mixed model with binomial structure in which the outcome variable was whether or not each individual that was ever detected during the initial learning experiment (N=409) subsequently met all of the criteria for participating and learning in all three experiments (i.e., a binary variable in which birds received a score of 1 if they: participated in the initial learning experiment and met the learning criterion in the initial learning experiment and participated in the first reversal and met the learning criterion in the first reversal and participated in the second reversal and met the learning criterion in the second reversal, and received a score of 0 if they failed to meet any of the above conditions). The reference levels for categorical factors are as follows: age (juvenile), sex (female), species (blue tit). Model estimates are calculated from a full model containing all variables. A stepwise elimination revealed no significant effects of any variable.

Supplementary Table 4. Results from generalized linear mixed models for factors influencing variation in learning speed.

| Experiment | fixed terms | estimate ± SE | *t* | *P* |
| --- | --- | --- | --- | --- |
| Initial learning | Intercept | 3.50±0.15 |  |  |
|  | Species | **-0.72±0.16** | **-4.53** | **<0.001** |
|  | Own feeder malfunctioning time (hrs) | **0.07±0.02** | **3.85** | **<0.001** |
|  | Other feeder malfunctioning time (hrs) | **0.07±0.01** | **5.31** | **<0.001** |
|  | Feeder location | **-1.55±0.17** | **-9.32** | **<0.001** |
|  | Age | -0.03±0.16 | -0.21 | 0.83 |
|  | Sex | -0.09±0.16 | -0.59 | 0.56 |
|  | Visit interval | -0.09±0.08 | -1.10 | 0.27 |
|  | Age*Sex | -0.19±0.33 | -0.56 | 0.58 |
|  | Age*Species | -0.07±0.34 | -0.21 | 0.84 |
|  | Sex*Species | -0.26 ±0.32 | -0.82 | 0.42 |
|  | Age*Sex*Species | 0.60±0.69 | 0.87 | 0.39 |
|  |  |  |  |  |
| Reversal learning 1 | Intercept | 3.47±0.14 |  |  |
|  | Own feeder malfunctioning time (hrs) | **0.12±0.04** | **3.15** | **0.002** |
|  | Other feeder malfunctioning time (hrs) | **0.06±0.02** | **3.05** | **0.003** |
|  | Feeder location | **-0.43±0.16** | **-2.70** | **0.008** |
|  | Number rewards after criterion in initial learning | 0.0011±0.00058 | 1.97 | 0.0504 |
|  | Age | -0.15±0.16 | -0.90 | 0.37 |
|  | ln(Learning speed in initial learning) | -0.06±0.05 | -1.16 | 0.25 |
|  | Sex | -0.18±0.16 | -1.16 | 0.25 |
|  | Social group treatment | 0.04±0.27 | 0.14 | 0.90 |
|  | Species | -0.21±0.17 | -1.27 | 0.20 |
|  | Visit interval | 0.02±0.08 | 0.30 | 0.77 |
|  | Age*Sex | -0.03±0.33 | -0.09 | 0.93 |
|  | Age*Species | -0.20±0.35 | -0.56 | 0.58 |
|  | Sex*Species | -0.43±0.32 | -1.34 | 0.18 |
|  | Age*Sex*Species | 0.40±0.70 | 0.57 | 0.57 |
|  |  |  |  |  |
| Reversal learning 2 | Intercept | 4.60±0.45 |  |  |
|  | Own feeder malfunctioning time (hrs) | **0.12±0.02** | **5.35** | **<0.001** |
|  | Other feeder malfunctioning time (hrs) | **0.04±0.01** | **3.00** | **0.003** |
|  | Feeder location | **-0.59±0.17** | **-3.50** | **<0.001** |
|  | Number rewards after criterion in first reversal learning | **-0.003±0.0009** | **-3.00** | **0.003** |
|  | Age | -0.17±0.17 | -1.00 | 0.32 |
|  | ln(reversal learning 1 speed) | 0.11±0.08 | 1.48 | 0.14 |
|  | Sex | -0.15±0.16 | -0.90 | 0.37 |
|  | Social group treatment | -0.86±0.48 | -1.79 | 0.13 |
|  | Species | -0.12±0.18 | -0.71 | 0.48 |
|  | Visit interval | -0.002±0.11 | -0.02 | 0.98 |
|  | Age*Sex | -0.10±0.34 | -0.28 | 0.78 |
|  | Age*Species | -0.14±0.37 | -0.38 | 0.70 |
|  | Sex*Species | 0.31±0.34 | -0.93 | 0.35 |
|  | Age*Sex*Species | -0.98±0.73 | -1.34 | 0.18 |

Learning speeds were natural log transformed. Estimates (calculated using restricted maximum likelihood), *t* values and *P* values are shown from separate models for each of the three experiments. Significant factors from the final model are shown in bold. The reference levels for categorical factors are as follows: age (juvenile), sex (female), species (blue tit), feeder location (centre), social group treatment (stable). Visit interval was standardized to have a mean of zero and standard deviation of one prior to model fitting. Sample sizes are 221 for initial learning, 198 for first reversal learning and 183 for second reversal learning. Learning speed is the number of visits to criterion, therefore positive effects represent slower learning.

Supplementary Table 5: Unadjusted and adjusted consistency values

|  | Blue tit, all experiments | | | Blue tit, reversals only | | | Great tit, all experiments | | | Great tit, reversals only | | |
| --- | --- | --- | --- | --- | --- | --- | --- | --- | --- | --- | --- | --- |
|  | r | SE | P | r | SE | P | r | SE | P | r | SE | P |
| Unadjusted | 0.014 | 0.039 | 0.40 | 0.138 | 0.085 | 0.07 | 0 | 0.04 | 0.5 | 0.284 | 0.112 | 0.008 |
| Adjusted | 0.09 | 0.056 | 0.06 | 0.115 | 0.087 | 0.13 | 0.009 | 0.05 | 0.5 | 0.282 | 0.113 | 0.014 |

Values correspond to the coefficient from a repeatability analysis with the independent variable learning speed (ln-transformed) and individual ID as a random effect, that was either unadjusted (only fixed effect was experiment ID), or adjusted (fixed effects: experiment ID, malfunctioning time of own feeder, malfunctioning time of other feeders, bias towards edge feeders (see below for description of bias measurements), bias towards neighbouring feeders and bias towards feeder assigned in previous experiment (this factor was included only for analyses that included reversal experiments only). Separate values were calculated for each species, and for all three experiments (initial discrimination, first reversal, second reversal) and for just the two reversal experiments.


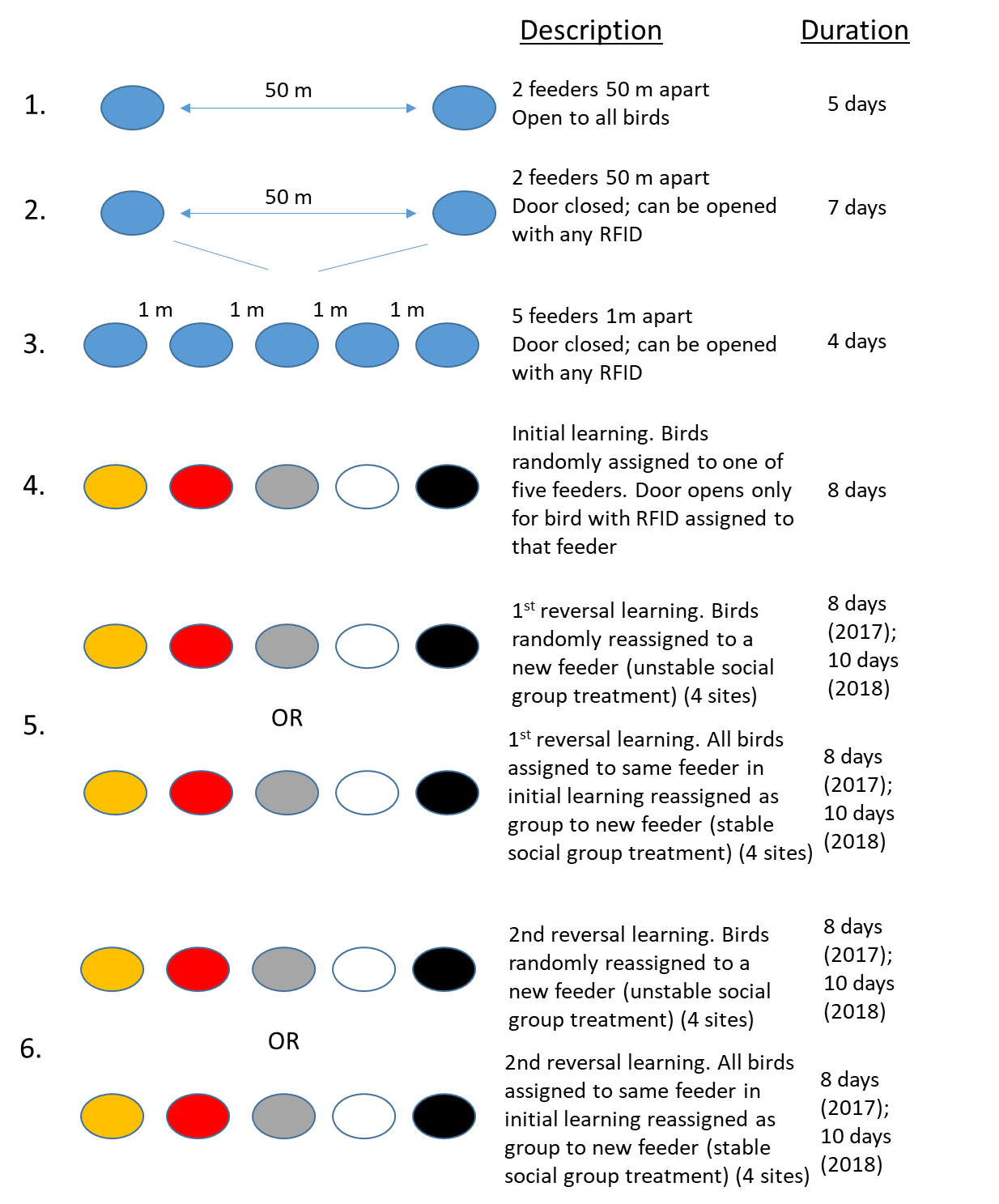


Supplementary Figure 1. Description of the experimental design. Each circle represents a feeder. Steps in the experimental design are depicted in chronological order from top (earliest) to bottom (latest); the duration of each step is given in the last column. The feeders are coloured differently during the learning stages to illustrate that each bird could only obtain food from one of them. Further description of each experimental phase is given in the main text.


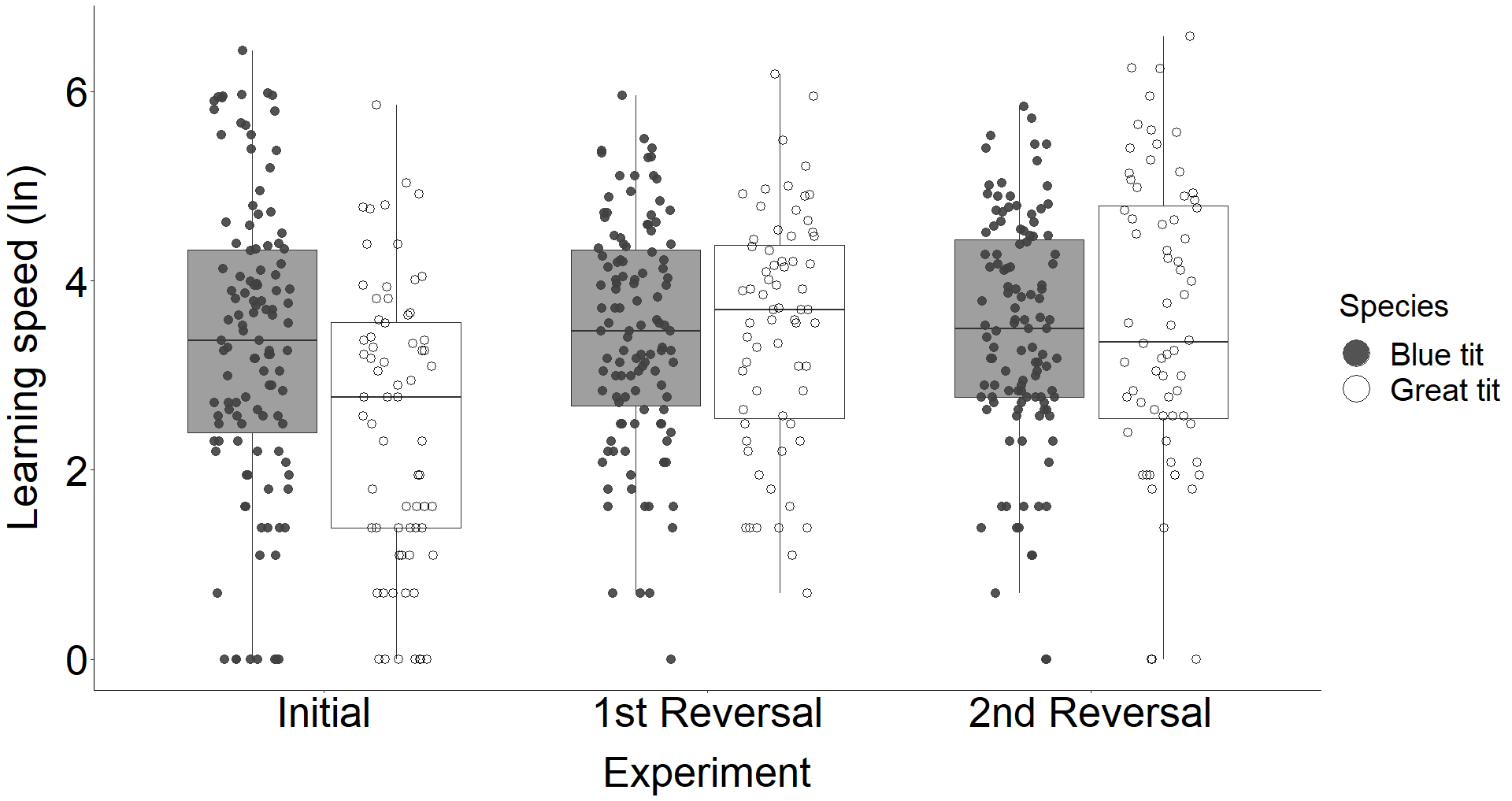


Supplementary Figure 2. Learning speeds (natural log transformed) for each species in each experiment. Boxplots (gray, blue tit; white, great tit) show median, interquartile range, and the whiskers extend up to 1.5 times the interquartile range. Points (filled, blue tit; open, great tit) represent learning speeds for each individual. Only those individuals (*N* = 183) that participated and met the learning criterion in all three experiments are included in this figure. Points have been jittered on the x-axis to reduce overlap. Analyses reported in the main text show that there was a statistically significant effect of species on learning speed in the initial learning experiment, but not in either of the two reversal learning experiments.
